# The biological behavior optimization of human periodontal ligament stem cells via preconditioning by the combined application of fibroblast growth factor-2 and A83-01 in in vitro culture expansion

**DOI:** 10.1186/s12967-019-1799-1

**Published:** 2019-02-28

**Authors:** Chunshu Zhang, Hongmei Guo, Chengzhe Yang, Qian Chen, Jiahui Huang, Lianlian Liu, Yu Zhang, Shanshan Jin, Aimei Song, Pishan Yang

**Affiliations:** 1 Department of Periodontology, School of Stomatology, Shandong University, 44 West Wenhua Road, Jinan, 250012 Shandong People’s Republic of China; 2 Shandong Provincial Key Laboratory of Oral Tissue Regeneration, Jinan, China; 3 Department of Oral and Maxillofacial Surgery, Qilu Hospital and Institute of Stomatology, Shandong University, Jinan, 250012 Shandong People’s Republic of China

**Keywords:** Periodontal ligament stem cells, Fibroblast growth factor-2, A83-01, Cell expansion, Osteogenic differentiation, Paracrine action

## Abstract

**Background:**

As the optimal source of seed cells in periodontal tissue engineering, periodontal ligament stem cells (PDLSCs) have always been researched to improve cell expansion due to their limited resource and spontaneous differentiation in vitro cultivation. Fibroblast growth factor-2 (FGF-2) has been proven to stimulate bone marrow mesenchymal stem cells (BMMSCs) proliferation and maintain their pluripotency when being added to the culture medium. As a small molecule inhibitor of transforming growth factor-beta receptors (TGF-βRs), A83-01 can also promote cell proliferation. Therefore, the aim of this study was to verify whether the combined application of FGF-2 and A83-01 could augment cell quantity and quality during in vitro culture.

**Methods:**

PDLSCs were preconditioned with A83-01, FGF-2, or their combination. A cell counting kit-8 (CCK8) assay, cell apoptosis assay, ALP activity assay, Alizarin Red S staining assay, RT-PCR assay, Western blot assay and ELISA were used to determine the sustained effects of different preconditioning strategies on the proliferation, apoptosis, stemness, osteogenic differentiation and paracrine action of PDLSCs.

**Results:**

The combined application of FGF-2 and A83-01 significantly augmented cell expansion, reduced cell apoptosis, magnified stemness expression, promoted later osteogenic differentiation and mineralization and increased paracrine action of PDLSCs compared with the control. Moreover, the combination presented significant advantages in enhancing proliferation, stemness expression and paracrine action over FGF-2 alone.

**Conclusions:**

The combined application of A83-01 and FGF-2 may be an improved strategy for PDLSCs biological behavior optimization in culture expansion and advantageous for reinforcing proliferation, stemness expression and cytokine secretion over FGF-2 alone.

## Background

Periodontitis is a kind of chronic progressive disease that is characterized by alveolar bone resorption and the loosening and loss of teeth. The main objective of periodontitis treatment is to achieve the regeneration of the functional periodontal tissue, in which the regeneration of bone plays a key role. Multiple strategies, including the bone graft material, bioactive molecule, guided tissue regeneration (GTR), etc., have been developed for this objective. Currently, PDLSCs-based tissue engineering is being studied worldwide. PDLSCs are a type of mesenchymal stem cell (MSCs) that are isolated from the root of the tooth and similar to other MSCs such as BMMSCs [1], dental pulp stem cells [2] and apical papilla stem cell [3], they have the ability of self-renewal and multipotent differentiation [4]. Sufficient evidences have demonstrated that PDLSCs can differentiate into osteoblast, cementoblast and fibroblast under proper culture condition in vitro while promoting the formation of new bone, functional periodontal ligament fiber and new cementum in vivo [5–10]. Therefore, PDLSCs are regarded as the optimal source of seed cells in periodontal tissue engineering [4]. However, MSCs including PDLSCs have some shortcomings, such as having limited sources and a loss of stemness while being cultured. Thus, the utility of MSCs as a cell-based therapy is limited [11, 12]. Additionally, increasing studies have shown that paracrine factors from MSCs are strongly associated with tissue regeneration and wound healing upon MSC transplantation [13, 14]. Paracrine effects of MSCs include immunomodulation, anti-apoptosis, angiogenesis, supporting the growth of cells and chemoattraction properties. Transplantation of conditioned medium (CM), which contains paracrine factors such as the insulin-like growth factor (IGF-1) and vascular endothelial growth factor (VEGF), has been reported to enhance wound healing in animal models [15]. This implies that the more paracrine factors MSCs, including PDLSCs, produce, the greater tissue regeneration and wound healing potentials of MSC-CM. Therefore, supplementing some ingredients into the culture medium to obtain a sufficient amount of PDLSCs that both have both the optimal potential of osteogenic differentiation and secret tissue regeneration-associated paracrine factors with fewer passages allows for periodontal regeneration treatment by transplantation of PDLSCs or PDLSC-CM.

FGF-2 has been proven to stimulate BMMSCs proliferation and maintain its pluripotency while being added to the culture medium [16]. Furthermore, our previous study has also found that preconditioning with FGF-2 for 48 h could enhance sustained proliferation and osteogenic differentiation capacity of BMMSCs [17]. As a small molecule inhibitor of TGF-βRs, A83-01 can promote cell proliferation by inhibiting SMAD2 phosphorylation and TGF-βR1 [18]. This signaling pathway can lead to cell cycle arrest by suppressing the expression of c-Myc and promoting expression of the cell cycle suppressor proteins P21 and P15 [19–21]. Based on these findings, we hypothesized that combined application of A83-01 and FGF-2 was an improved strategy for PDLSC biological behaviour optimization in culture expansion over FGF-2 alone. The purpose of this study was to evaluate the sustained effect of combined A83-01 and FGF-2 preconditioning on proliferation, apoptosis, stemness, potential of osteogenic differentiation and paracrine of PDLSCs.

## Methods

### Cell culture

Twenty human premolars from 10 orthodontic individuals and 5 impacted third molars from 5 patients (aged 19–29 years) were collected and stored in Dulbecco’s modified Eagle’s medium (DMEM; HyClone, Logan, UT, USA) with penicillin G (100 U/ml) and streptomycin (100 μg/ml). All experiments were conducted with strict adherence to the guidelines of the Institutional Review Board of Shandong University Stomatological College. Periodontal ligament tissue was isolated from the middle part of the root surface with surgical blades, samples from different individuals were pooled [4] and digested with collagenase I (3 mg/ml, Sigma-Aldrich, St. Louis, MO, USA) and dispase II (4 mg/ml; Sigma-Aldrich) for 50 min. Then, the cells were cultured with alpha-modified Eagle’ medium (α-MEM; Invitrogen, USA) containing 20% foetal bovine serum (FBS, Invitrogen) at 37 °C under 5% carbon dioxide. The primary cells were cloned with the limiting-dilution method to achieve the purified stem cells according to our previously reported method [22]. Then, the stem cells were cultured in 10 CM cell culture dish using the same medium supplemented with 10% serum. The medium was changed and the non-adherent cell removed every 3 days. Cells were passaged to the 3rd generation and were ready for the following experiments.

### Optimal concentration determination of A83-01 in term of cell proliferation promotion

The digestive cells were resuspended in maintenance medium (DMEM containing 10% foetal bovine serum, 100 U/ml penicillin G and 100 μg/ml streptomycin, HyClone, USA), inoculated in 96-well plate (1 × 10^3^ cells/well) and cultured with different concentrations of A83-01 (0, 0.5, 1, 5, 10, 50, 100 μM). After 48 h the original solution was removed and 100 μl culture medium containing 10 μl CCK 8 (Dojindo Laboratories, Kumamoto, Japan) was added to each well according to the manufacturer’s instructions. The plate was then incubated for another 2 h and the absorbance at 450 nm wavelength was measured with the microplate reader (SPECTRO-star Nano; BMG Labtech, Ortenberg, Germany).

### Cell preconditioning by FGF-2 or/and A83-01

All cells were divided into four groups: the control group, FGF-2-preconditioning group (10 ng/ml FGF-2, 100-18B; PEPROTECH, USA), A83-01-preconditioning group (5 μM A83-01, 9094360; PEPROTECH, USA) and FGF-2 + A83-01-preconditioning group (10 ng/ml FGF-2 + 5 μM A83-01). Each group of cells was incubated for 48 h in 6-well plate (8 × 10^4^ cells/well) containing maintenance medium, 1% foetal bovine serum, 100 U/ml penicillin G, 100 μg/ml streptomycin and designated medication according to above grouping with medium changed every day.

### Proliferation assay of preconditioned cells

After 48 h of preconditioning culture, cells were washed three times with phosphate buffered solution (PBS) and re-cultured in maintenance medium with only 10% FBS until the cells obtained 80% confluence. Then, the cells were collected and seeded in 96-well plate (1 × 10^3^ cells/well) for CCK 8 assay. The absorbance was measured at scheduled time points. The morphology of the pre/unpreconditioned PDLSCs re-cultured for 24 h in the maintenance medium was observed under inverted microscope (Olympus, Tokyo, Japan).

### Apoptosis analysis of preconditioned cells

The preconditioned PDLSCs were collected and cultured in 6-well plates at a density of 8 × 10^4^ cells and were well supplemented with maintenance medium. Three days later, cells were trypsinized, washed and resuspended in 1 ml PBS. For apoptosis assay, cells collected from each well were resuspended with 500 μl 1× Binding Buffer, 5 μl Annexin V and 10 μl of propidium iodide (MultiSciences (Lianke) Biotech Co, Ltd, Hangzhou, china) were added to each Eppendorf tube to stain the cells with and without intact cellular membrane for 10 min at room temperature. In the end, the rate of cell apoptosis was analysed by flow cytometry (CytoFLEX; Beckman Coulter, Brea, CA, USA).

### ALP activity detection of preconditioned cells

The preconditioned PDLSCs were cultured in 96-well plate at a density of 2 × 10^3^ cells/well with maintenance medium containing 10% FBS. When the cells adhered up to 80%, medium was changed into osteogenic inductive medium (10^−8^ M dexamethasone (Sigma-Aldrich, USA), 10 mM β-glycerophosphate (Sigma-Aldrich, USA) and 50 ng/ml ascorbic acid (Sigma-Aldrich, USA) in DMEM-F12 containing 10% FBS). Seven days later, 1% TritonX-100 (Solarbio) was added for cell lysis and intracellular proteins were collected via supersonic splitting and centrifuging. Then, a BCA Protein assay kit (Nanjing Jiancheng Bioengineering Institute, Nanjing, China) was used to detect the total protein concentration and ALP kits (Nanjing Jiancheng Bioengineering Institute, Nanjing, China) were used to assay alkaline phosphatase (ALP) activity according to the manufacturer’s instructions. At last, the absorbance was measured at 520 nm wavelength.

### Alizarin red staining

The preconditioned PDLSCs were cultured in 6-well plates with osteogenic inductive medium. Twenty-eight days later, the cells were washed three times with PBS and fixed in 4% paraformaldehyde for 15 min. Then, the cells were washed for another three times and stained with Alizarin red (PH 4.2; Sigma-Aldrich) for 5 min. Then, the formation of mineralized nodule was observed under inverted microscope.

### RT-PCR analysis for gene expression of stemness and osteogenic markers

To analyse gene expression of stemness and osteogenic markers, the preconditioned PDLSCs were respectively cultured in medium supplemented only with 10% FBS and osteogenic inductive medium. The total RNA of the cells was extracted with Trizol. After being quantified, the mRNA was reverse transcribed to cDNA with PrimeScript™ RT reagent Kit and gDNA Eraser (Takara). Each reactive mixture contained 1 μl cDNA, 5 μl SYBR Premix Ex Taq II (Tli RNaseH Plus; Takara), 3.6 μl sterile threefold-distilled water and 0.2 μl forward primer and reverse primer. Finally, the reaction was performed on a Light Cycler Roche 480 II Real-Time PCR System (Roche, Basel Switzerland) in triplicate. The primer sequences were showed in Table 1, and the gene expression level was normalized to GAPDH before statistical analysis.

**Table 1:** Primer sequences for qRT-PCR

Gene	Forward primer (5′-3′)	Reverse primer (5′-3′)
GAPDH	GCACCGTCAAGGCTGAGAAC	TGGTGAAGACGCCAGTGGA
ALP	ATGGGATGGGTGTCTCCACA	CCACGAAGGGGAACTTGTC
RunX2	TCCACACCATTAGGGACCATC	TGCTAATGCTTCGTGTTTCCA
OPN	TCCTAGCCCCACAGACCCTT	CACACTATCACCTCGGCCAT
c-Myc	CCCGCTTCTCTGAAAGGCTCTC	CTCTGCTGCTGCTGCTGGTAG
Nanog	CAGAAGGCCTCACACCTAC	ATTGTTCCAGGTCTGGTTGC

### Western blot analysis for protein expression of osteogenic markers

The preconditioned PDLSCs were cultured with an osteogenic inductive medium for 3, 7 and 14 days. The cells were then collected and washed 3 times with ice-cold PBS. RIPA lysis containing 1% PMSF (Solarbio) was used to extract proteins. Then, the mixtures were centrifuged at 12,000*g* for 15 min. The protein samples were treated with the same kit used in an ALP detection assay and the protein concentration was measured with microplate reader. Lysates were denatured at 100 °C for 5 min with an SDS-PAGE loading buffer added to them. The samples and BSA markers were loaded on a 10% SDS-PAGE gel and then transferred to PVDF membranes (GE Amersham, Fairfield, CT, USA). Membranes were blocked in 5% non-fat dry milk for 1 h and the primary antibodies were blotted overnight at 4 °C as follows: rabbit anti-ALP antibody (1:500, ab108337; Abcam, Cambridge, UK), rabbit anti-Runx2 antibody (1:2000, ab23981; Abcam) and rabbit anti-OPN antibody (1:1000, ab8448; Abcam). Subsequently, the membranes were incubated with secondary antibodies (1:20,000, ab150077; Abcam) for 1 h and then washed with tris-buffered saline with Tween 20 (TBST) three times. Chemiluminescence reagents (Millipore) were used for the development. The images were quantitatively analysed with Image J software (NIH, Bethesda, Maryland, USA). Each protein expression level was normalized to GAPDH before statistical analysis.

### ELISA

After preconditioning, all cells were cultured in ordinary culture medium for another 72 h. The collected supernatant was centrifugated for 15 min and then injected into a 96-well with three duplications for each group. All procedures were conducted strictly according to the specifications of the ELISA kit (Dakewe Biotech Co. Ltd. Beijing, China). The absorbance was measured at a 450 nm wavelength.

### Statistical analysis

Data were collected and expressed as the mean ± standard error of the mean (S. E. M.). Differences between groups were analysed using the one-way ANOVA through SPSS 19.0 (IBM, Armonk, NY, USA). Statistical probability of p < 0.05 was considered significant.

## Results

### Both A83-01 and FGF-2 preconditioning promoted the proliferation of PDLSCs, and their combination had a significantly superimposed effect

**Fig. 1:**
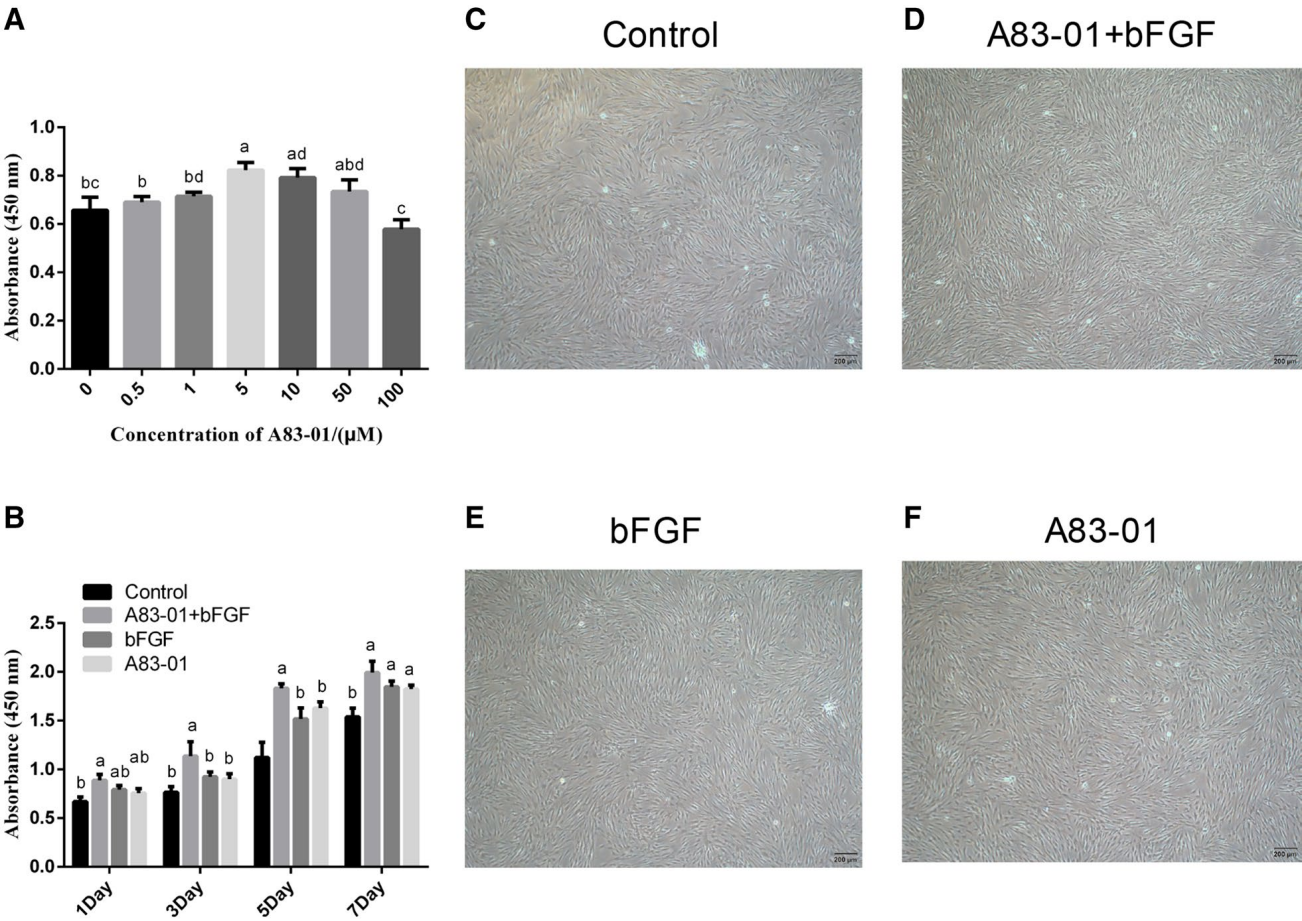
Effect of different concentrations of A83-01 and different preconditioning strategies on PDLSC proliferation. **A** PDLSCs were treated with 0, 0.5, 1, 5, 10, 50 and 100 μM A83-01 for 48 h. The proliferation activity of PDLSCs was analysed with CCK8 kit. **B** Proliferation activity of preconditioned groups and a negative control group after 1,3, 5 and 7 days of cultivation in a medium only supplemented with 10% FBS. **C**–**F** Morphological observation of pre/non-preconditioned PDLSCs after 24 h of re-culture. (No matching letters (e.g.: a, b, c, d) above any two column charts represents a significant difference between these two groups (*p *< 0.05), while the appearance of any matching letters (e.g.: a, a) means that there is no significant difference among groups (*p *> 0.05). Data are expressed as mean ± SD)

First, the optimal concentrations of A83-01 for PDLSC proliferation were determined by the CCK8 assay. The results showed that the proliferation of PDLSCs preconditioned with 5 and 10 μM A83-01 was higher than that of the control group (*p *< 0.05), with a peak at 5 μM (Fig. 1A). Then, PDLSCs were preconditioned by 5 μM A83-01 or 10 ng/ml FGF-2 or their combination for 48 h, and the preconditioned PDLSCs were re-cultured with the maintenance medium and the cell proliferation activity was measured via CCK8 assay. The results revealed that, compared with the control, the proliferative capacity of PDLSCs was significantly enhanced after being preconditioned with 10 ng/ml FGF-2 or 5 μM A83-01 (at day 5 and 7) and the combination of A83-01 and FGF-2 performed better in promoting the proliferation of PDLSCs than the control group (at all time points) and single stimulation groups (at day 3 and 5) (*p *< 0.05) (Fig. 1B). Morphologically, no obvious differences were observed among the four groups, except for the cell number (Fig. 1C–F).

### A83-01 and FGF-2 preconditioning or their combination decreased the apoptosis rate of PDLSCs

**Fig. 2:**
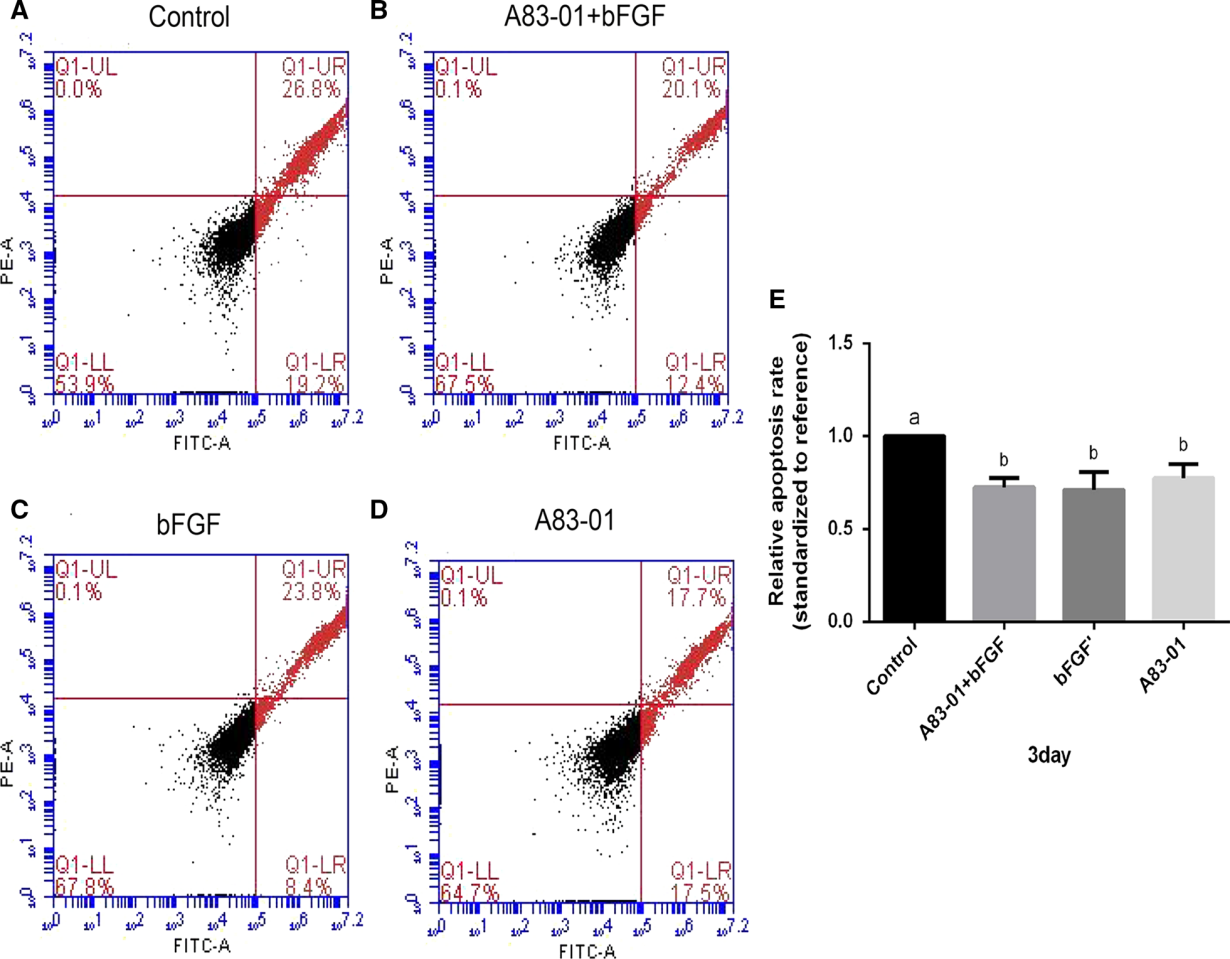
Effect of different preconditioning strategies on apoptosis of PDLSCs. Preconditioned and non-preconditioned PDLSCs were collected and passaged to the 4th generation. After 3 days of cultivation only supplemented with 10% FBS, the cells were collected and stained with annexin V-FITC and propidium iodide (PI). Apoptosis was tested by flow cytometry. **A**–**D** Apoptosis images of four groups. **E** Significant difference could be found between control group and pretreatment groups while no significant difference was noted among three pretreatment groups. (No matching letters (e.g.: a, b) above any two column charts represents a significant difference between these two groups (*p *< 0.05), while the appearance of any matching letters (e.g.: a, a) means that there is no significant difference among groups (*p *> 0.05). Data are expressed as mean ± SD)

The apoptosis rate of PDLSCs was analysed by flow cytometry with Annexin V and PI, which could differentiate the early apoptosis process from the late one. In our research, the apoptosis rates of the three preconditioning groups were obviously lower than the rate of the control group (*p *< 0.05), while no significant differences were found among three preconditioning groups (Fig. 2A, B). This proved that both A83-01 and FGF-2 could efficiently decrease the apoptosis of PDLSCs, but their combination exerted no significantly superimposed effect.

### Both A83-01 and FGF-2 preconditioning or their combination maintained or even enhanced osteogenic differentiation and mineralizing potentials in PDLSCs

**Fig. 3:**
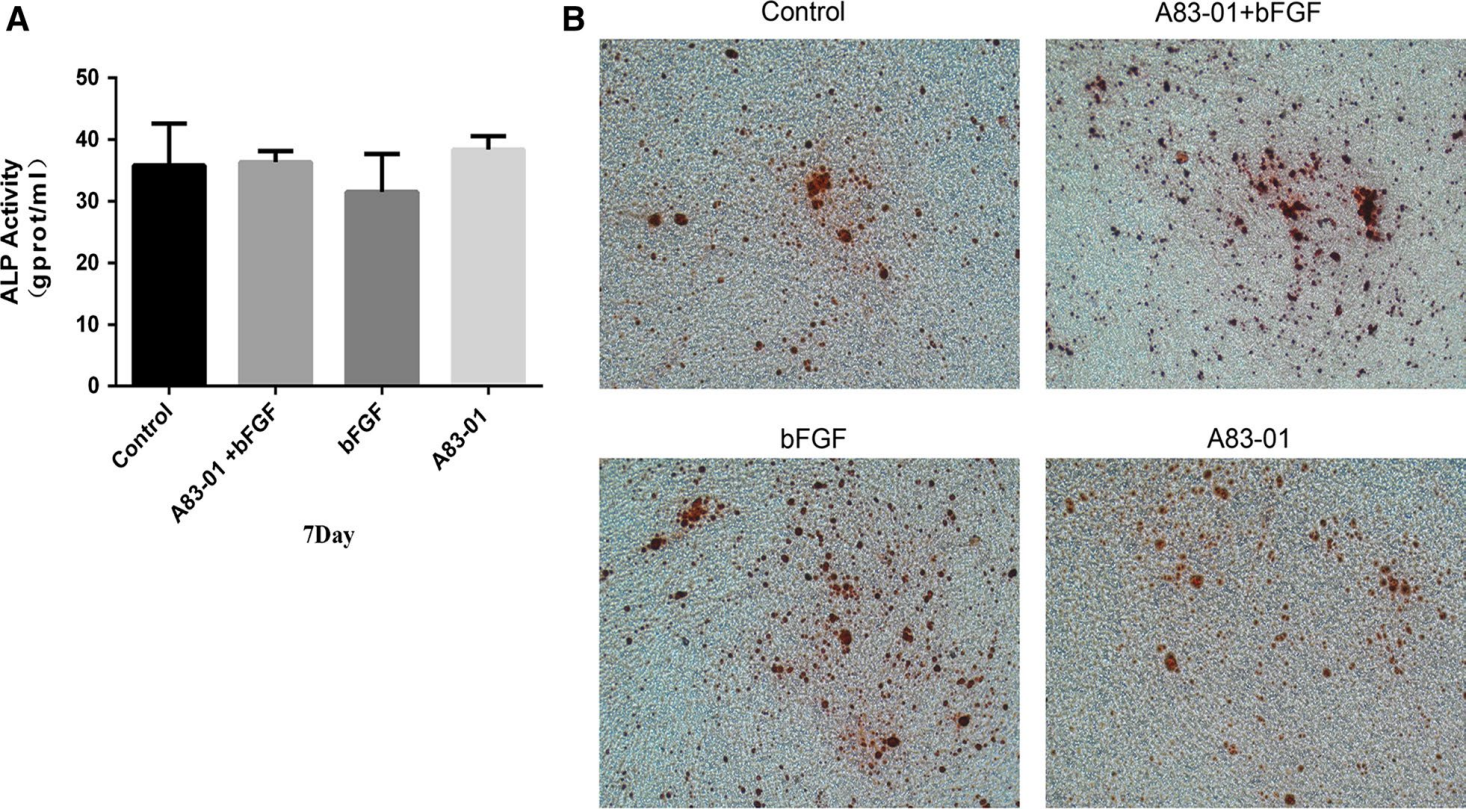
Effect of different preconditioning strategies on ALP expression and calcium nodules formation of PDLSCs. PDLSCs were treated in a culture medium with or without stimulations for 48 h and then cultured in an osteogenic inductive medium without stimulations for 7 and 21 days. **A** 7 day later, cells were collected for the ALP activity assay. **B** 21 day later, Alizarin Red staining was performed to detect the formation of mineralized nodules

As a marker of early osteoblastic differentiation, the ALP activities of the cells in three preconditioning groups and the control group was measured after 7 days of osteogenic induction. The result showed that there was no remarkable difference in ALP activity among the four groups (Fig. 3A). After 21 days of osteogenic induction, the cells were stained by Alizarin Red. It seemed that the three preconditioning groups were able to form a similar number of calcified nodules as the control group (Fig. 3B).

**Fig. 4:**
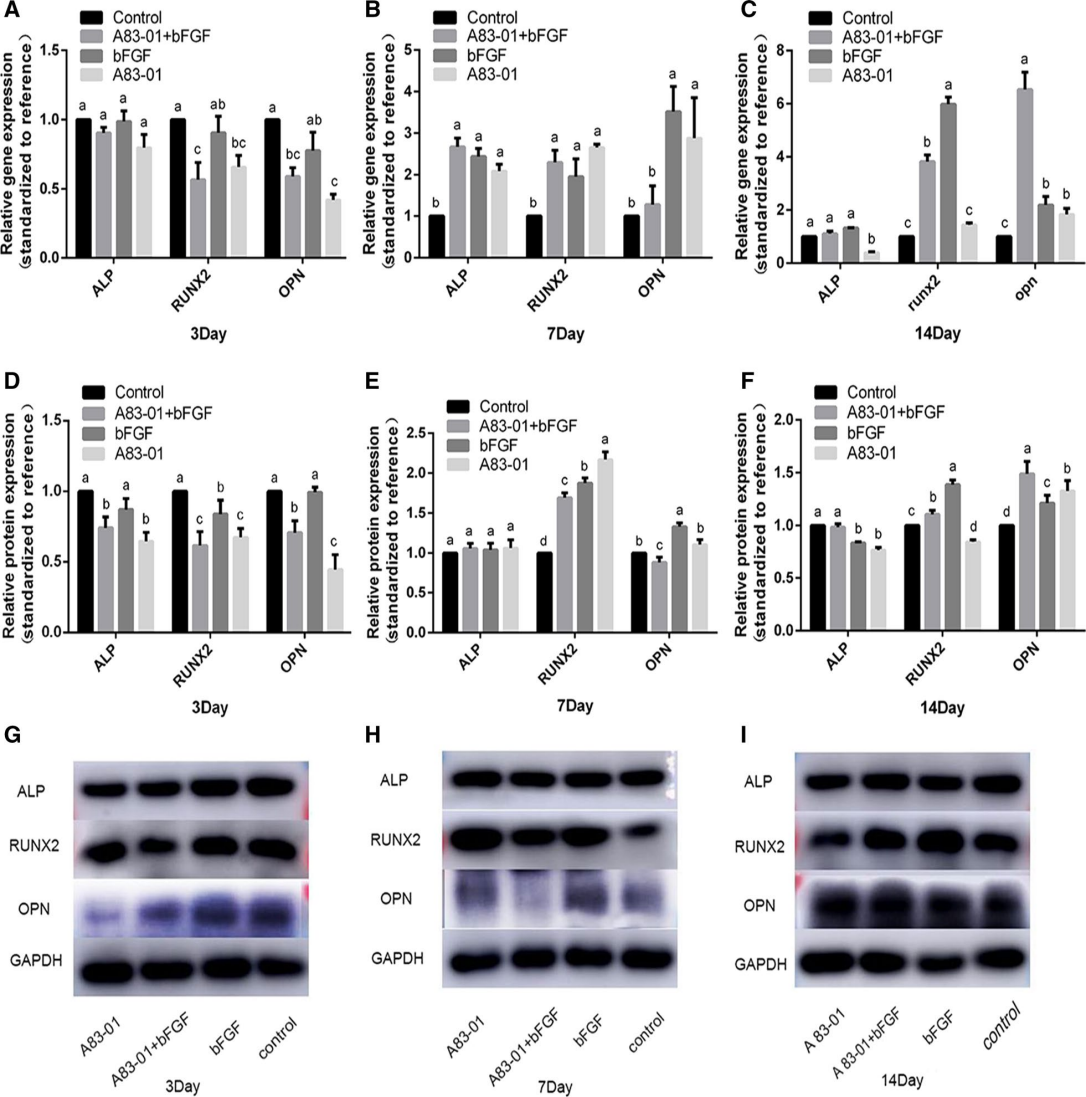
Detection of the alteration of osteogenesis-related indexes at the gene and protein levels. Pre/non-preconditioned PDLSCs were cultured in an osteogenic inductive medium without stimulations. **A**–**I** After 3, 7, 14 days of osteogenic induction, the RNA and protein were collected for RT-PCR and Western blot assay. (No matching letter (e.g.: a, b, c, d) above any two column charts represents a significant difference between these two groups (*p *< 0.05), while the appearance of any matching letters (e.g.: a, a) means that there is no significant difference among groups (*p *> 0.05). Data are expressed as mean ± SD)

The qRT-PCR assay showed that at day 3, though the mRNA expression of the ALP in the three preconditioning groups and Runx-2 and OPN in FGF-2 preconditioning group were not significantly changed, A83-01 or the combination preconditioning significantly reduced Runx-2 and OPN mRNA expression compared with the control group (*p *< 0.05) (Fig. 4A). At day 7, three preconditioning strategies all increased mRNA expression of ALP, Runx-2 and OPN (*p *< 0.05), although no significant difference in OPN expression was found between the combination preconditioning and the control group (Fig. 4B). At day 14, the mRNA expression levels of ALP, Runx-2 and OPN were maintained (ALP in FGF-2 and the combination preconditioning groups and Runx-2 in the A83-01 preconditioning group) or improved (Runx-2 in FGF-2 and combination preconditioning groups and OPN in three preconditioning groups) compared with the control group (*p *< 0.05) (Fig. 4C).

The Western bolt assay showed that after 3 days of induction, compared with the control group, the A83-01 and combination preconditioning groups showed decreased protein expression of ALP, Runx-2 and OPN, of which the former was more obvious, while the FGF-2 preconditioning group maintained ALP and OPN but reduced Runx-2 expression (*p *< 0.05) (Fig. 4D, G). At day 7, there was no significant difference in the expression of ALP among the four groups. The Runx-2 expression in all the preconditioning groups was significantly higher than that in control group. The expression of OPN was higher in the FGF-2 preconditioning group, unchanged in the A83-01 preconditioning group, and lower in the combination preconditioning group than in the control group (*p *< 0.05) (Fig. 4E, H). At day 14, the expression of ALP in the combination preconditioning group essentially remained unchanged, whereas that in the FGF-2 group and A83-01 group unexpectedly decreased compared with the control group. The Runx-2 protein expression in the combination group was higher than the control group but lower than that in the FGF-2 group (*p *< 0.05). The OPN protein expression in FGF-2 group and A83-01 group was higher than that in the control group, but significantly lower than that in the combination group (Fig. 4F, I).

Taken together, though there were unexpected results, for example, an inconsistent expression of mRNA and protein at three time points or decreased protein expression of ALP in the FGF-2 group and A83-01 group at day 14 compared with the control group, the comprehensive tendency suggested that both A83-01 and FGF-2 preconditioning or their combination were inhibited at an early period and were maintained or even enhanced at medium—later stage osteogenic differentiation and mineralizing potentials in PDLSCs.

### Both A83-01 and FGF-2 preconditioning promoted the expression of stemness genes in PDLSCs and their combination exerted a significantly superimposed effect

**Fig. 5:**
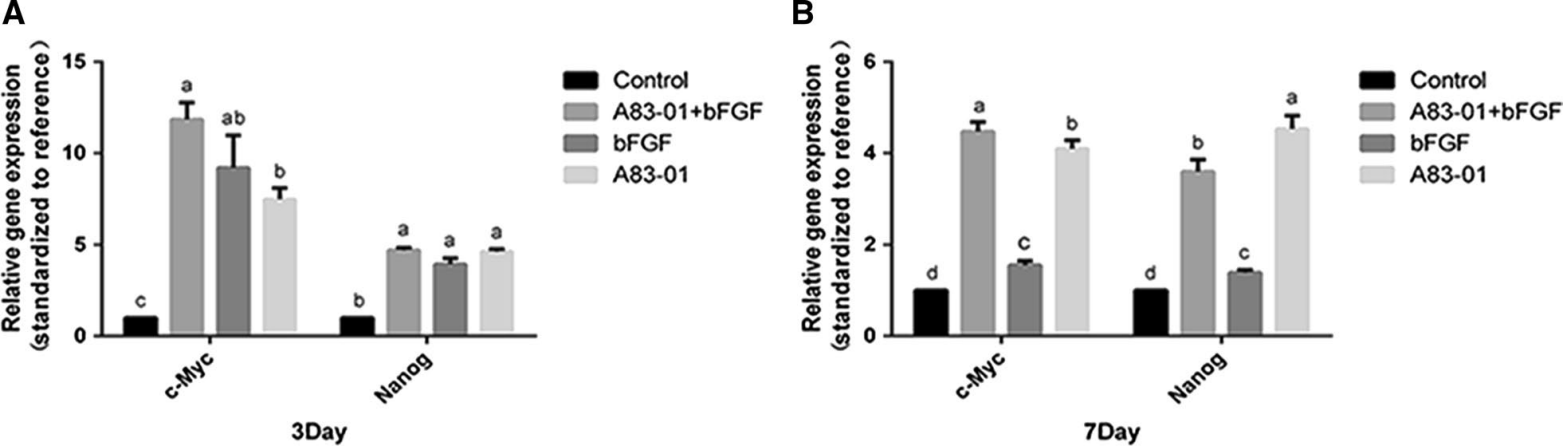
Effect of different preconditioning strategies on stemness maintenance of PDLSCs. Fourth generation PDLSCs preconditioned by various treatments were cultured in the same culture medium for 3 and 7 day. Cells were collected for RT-PCR. The gene expression of c-Myc and Nanog was measured on Light Cycler Roche 480 II Real-Time PCR System. **A** Comparision of gene expression of c-Myc and Nanog among different groups after 3 day culture. **B** Comparision of gene expression of c-Myc and Nanog among different groups after 7 day culture. (No matching letters (e.g.: a, b, c, d) above any two column charts represents a significant difference between these two groups (*p *< 0.05), while the appearance of any matching letters (e.g.: a, a) means that there is no significant difference among groups (*p *> 0.05). Data are expressed as mean ± SD)

The gene expression of c-Myc and Nanog was detected to determine whether the combination of A83-01 and FGF-2 effected the stemness of PDLSCs. The results showed that 3 and 5 days after preconditioning, the three preconditioning strategies significantly enhanced the expression of c-Myc and Nanog. Moreover, the combination preconditioning had a significantly stronger effect on the c-Myc expression than A83-01 preconditioning at day 3 and on the expression of c-Myc and Nanog than FGF-2 preconditioning at day 7 (*p *< 0.05) (Fig. 5A, B).

### Both A83-01 and FGF-2 preconditioning or their combination dramatically augmented cytokine secretion in PDLSCs

**Fig. 6:**
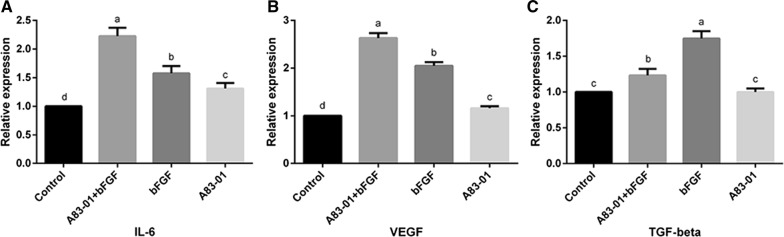
Effect of different preconditioning strategies on the paracrine potential of PDLSCs. Cells that underwent pre/non-preconditioning were cultivated in an ordinary culture medium for 72 h. The supernatant was then collected for ELISA. **A**–**C** The expression of IL-6, VEGF and TGF-β of different groups were measured with a microplate reader. [No matching letter (e.g.: a, b, c, d] above any two column charts represents a significant difference between these two groups (*p *< 0.05), while the appearance of any matching letters (e.g.: a, a) means that there is no significant difference among groups (*p *> 0.05). Data are expressed as mean ± SD)

We examined the concentrations of IL-6, VEGF and TGF-β in culture supernatant to explore whether the three preconditioning strategies would affect the paracrine efficiency of PDLSCs. The results revealed that in all three preconditioning groups there was significant elevation in the concentrations of IL-6 and VEGF. Moreover, the highest levels of these two factors were observed in the combination preconditioning group (*p *< 0.05) (Fig. 6A, B). Additionally, the concentrations of TGF-β in the FGF-2 and combination preconditioning groups were significantly higher than those in the control group and A83-01 group (*p* < 0.05) (Fig. 6C).

## Discussion

PDLSCs have been regarded as the optimal source of seed cells [3] and stem cell-conditioned medium [23] in periodontal tissue engineering. However, it is crucial to isolate and expand them in vitro before using them for clinical practice. The limited sources, loss of stemness and replicative senescence while being cultured limit their wide utility as a cell-based therapy [11, 12]. It has been reported that the replicative senescence of MSCs includes a progressive loss of proliferation potential and a declining differentiation ability [24]. Therefore, supplementing some ingredients into the culture medium to achieve more qualified PDLSCs during fewer passages and to make MSCs secret more tissue regeneration associated paracrine factors during the in vitro culture expansion allows for periodontal regeneration treatment by transplantation of PDLSCs or PDLSC-CM. The present study demonstrated that the combination of A83-01 and FGF-2 significantly augmented cell expansion, reduced cell apoptosis, magnified stemness expression, promoted later osteogenic differentiation and mineralization and increased paracrine action of PDLSCs compared with the control. Moreover, it presented significant advantages in proliferation, stemness expression and paracrine action over FGF-2 or A83-01 preconditioning.

As mentioned above, the loss of stemness, replicative senescence and decreased proliferation ability with prolonged culture time are typical properties of MSCs. Thus, maintaining stemness and promoting proliferation are important for stem cell expansion culture. In addition to promoting angiogenesis, nerve regeneration, migration of MSCs and chemotactic activity, FGF-2 has been demonstrated to regulate cell proliferation and sustain the self-renewal ability [25, 26]. The present study revealed once again that FGF-2 preconditioning could enhance proliferation, increase stemness marker expression and reduce apoptosis of PDLSCs. Moreover, the combination of FGF-2 and A83-01 played a superimposed role in increased proliferation and stemness marker expression.

TGF-β is a multifunctional cytokine that plays a regulatory role in cell proliferation, apoptosis, differentiation and wound healing [27]. The biological effects of TGF-β1 could only be achieved through the signal transduction of receptors on the cell membrane surface. Studies showed that both TGF-βR1 and TGF-βR2 were involved in the activation of TGF-β1/Smads signal transduction pathway which would inhibit cell proliferation [20, 21]. Therefore, inhibition of TGF-βRs may contribute to stem cell expansion. A83-01 is a selective inhibitor of TGF-βRs ALK4, 5, 7, which inhibits Smads2 phosphorylation. Studies have already demonstrated that self-renewal and proliferation of human induced pluripotent stem cells could be maintained after being treated by A83-01 [28]. This may be possible mechanism by which the combination of FGF-2 and A83-01 plays a superimposed role in proliferation and stemness expression promotion.

Osteoblastic differentiation is one of the main mechanisms by which PDLSCs promote periodontal regeneration. Thus, for the use of MSCs or MSC-CM in regenerative therapy, a suitable culture method that allows for the production of a large populations of cells and maintains and even enhances their osteogenic differentiation potential and paracrine effect should be established. FGF-2 has been proven to promote bone regeneration in fracture healing [29, 30] and in cranial [31], long [32], or periodontal bone defects [33]. FGF-2 expanded BMSCs presented significantly increased clonogenic growth, decreased percentage of alkaline phosphatase (ALP) activity positive colonies, and an enhanced in vivo osteogenic potential [25]. Similarly, in our previous studies FGF-2-preconditioning has been proven to accelerate the osteoblastic differentiation of BMMSCs [17, 26]. In this study, though there were unexpected results, for example, inconsistent expression of mRNA and protein at three time points or decreased protein expression of ALP in the FGF-2 group and A83-01 group at day 14 compared with the control group, the comprehensive tendency suggested that both A83-01 and FGF-2 preconditioning were inhibited at the early period and were maintained or even enhanced at medium—later stage osteogenic differentiation and mineralizing potentials in PDLSCs. However, their combination did not exert an unambiguous additive effect.

Paracrine is a vital means of communication among cells [34, 35]. As the research proved, MSCs could mediate migration, proliferation and apoptosis of surrounding cells via the paracrine pathway [36–38]. Moreover, some scholars even argued that instead of directly transforming into a tissue-related cell type, transplanted MSCs actually promote the proliferation and differentiation of autologous MSCs by regulating the microenvironment of the lesion site [39, 40]. Among the paracrine factors, VEGF, TGF-β and IL-6 play key roles in tissue repair, which participate in leukocyte infiltration, angiogenesis, collagen accumulation, lymphangiogenesis, osteogenic differentiation, recruiting MSCs, etc. during wound healing [40–42]. Our results revealed that in all three preconditioning groups there was significant elevation in concentrations of IL-6 and VEGF.

Moreover, the highest levels of these two factors were observed in the combination preconditioning group. Additionally, the concentrations of TGF-β in FGF-2 and combination preconditioning group were significantly higher than those in the control group and the A83-01 group.

## Conclusion

In summary, for the use of MSCs or MSC-CM in regenerative therapy, a suitable culture method that allows for the production of a large populations of cells and maintains and even enhances their osteogenic differentiation potential and paracrine effect should be established. We first proved that the combined application of A83-01 and FGF-2 significantly augmented cell expansion, reduced cell apoptosis, magnified stemness expression, promoted later osteogenic differentiation and mineralization and increased the paracrine action of PDLSCs compared with the control. Moreover, it presented significant advantages in proliferation, stemness expression and paracrine action over FGF-2 or A83-01 preconditioning. These results may provide a new method for the culture expansion of PDLSCs, and the cell properties exhibited in this study need to be testified in PDLSCs and PDLSC-CM-based periodontal regeneration in vivo research.

## Abbreviations

PDLSCs: periodontal ligament stem cells
FGF-2: basic fibroblast growth factor
BMMSCs: bone mesenchymal stem cells
IGF: insulin-like growth factor
VEGF: vascular endothelial growth factor
TGF-β: transforming growth factor-β
IL-6/10: interleukin-6/10
PBS: phosphate buffer solution
ALP: alkaline phosphatase
Fig: figure
